# Tbx2 and Tbx3 Regulate the Dynamics of Cell Proliferation during Heart Remodeling

**DOI:** 10.1371/journal.pone.0000398

**Published:** 2007-04-25

**Authors:** Inês Ribeiro, Yasuhiko Kawakami, Dirk Büscher, Ángel Raya, Joaquín Rodríguez-León, Masanobu Morita, Concepción Rodríguez Esteban, Juan Carlos Izpisúa Belmonte

**Affiliations:** 1 Gene Expression Laboratory, The Salk Institute for Biological Studies, La Jolla, California, United States of America; 2 Unidade de Desenvolvimento, Instituto Gulbenkian de Ciência, Oeiras, Portugal; Baylor College of Medicine, United States of America

## Abstract

**Background:**

The heart forms from a linear tube that is subject to complex remodeling during embryonic development. Hallmarks of this remodeling are the looping of the heart tube and the regionalization into chamber and non-chamber myocardium. Cardiomyocytes in the future chamber myocardium acquire different cellular and physiological characteristics through activation of a chamber-specific genetic program, which is in part mediated by T-box genes.

**Methodology/Principal Finding:**

We characterize two new zebrafish T-box transcription factors, *tbx3b* and *tbx2a*, and analyze their role during the development of the atrioventricular canal. Loss- and gain-of-function analyses demonstrate that *tbx3b* and *tbx2a* are necessary to repress the chamber-genetic program in the non-chamber myocardium. We also show that *tbx3b* and *tbx2a* are required to control cell proliferation in the atrioventricular canal and that misregulation of cell proliferation in the heart tube influences looping. Furthermore, we characterize the heart phenotype of a novel *Tbx3* mutation in mice and show that both the control of cell proliferation and the repression of chamber-specific genetic program in the non-chamber myocardium are conserved roles of Tbx3 in this species.

**Conclusions/Significance:**

Taken together, our results uncover an evolutionarily conserved role of Tbx2/3 transcription factors during remodeling of the heart myocardium and highlight the importance of controlling cell proliferation as a driving force of morphogenesis.

## Introduction

The T-box (Tbx) family of transcription factors is represented in all metazoans [1] and is characterized by a highly conserved DNA binding domain, the T-box domain. Several Tbx genes are expressed during embryonic development in specific domains and have been implicated in the formation of a variety of organs, including the heart. The existence of several human congenital syndromes caused by disruption of human *TBX* genes, demonstrates their importance in morphogenesis. The Holt-Oram syndrome is caused by *TBX5* haploinsufficiency and is characterized by heart and hand anomalies [2]. Mutations in *TBX1* have been implicated in the Velocardiofacial or DiGeorge syndrome in which congenital heart defects are present [3]. Several *Tbx* genes are expressed in the heart in discrete and overlapping regions and have been shown to be important for the development of such complex organ.

The embryonic linear heart tube undergoes complex remodeling, including looping and regionalization, to form the final vertebrate multichambered heart. Initially, the primary myocardium is morphologically homogenous throughout the extent of the linear heart tube. Concomitantly with the beginning of looping, the heart tube becomes regionalized into chamber myocardium, the atria and the ventricles, and non-chamber myocardium, the outflow tract (OFT), the atrioventricular canal (AVC), the inflow tract (IFT) and the inner curvatures. The chamber myocardium becomes fast conducting myocardium whereas the non-chamber myocardium retains slow conduction, thereby participating in the establishment of synchronized beating in the embryonic heart [4]. Additionally, the myocardium of the future chambers starts to grow out of the primitive heart tube during looping. Consequently, the non-chamber myocardium forms constrictions in the heart tube, such as the AVC, that prevent backflow of blood before valve formation. A variety of chamber and non-chamber specific genes have been characterized. The expression of chamber markers, such as atrium natriuretic factor (*Anf*), connexin 40 and 43 (*Cx40, Cx43*), *Chisel* and bone morphogenetic protein 10 (*Bmp10*), comes on concomitantly with the start of chamber growth in the mouse heart tube [5], [6]. The myocardium fated to become non-chamber myocardium expresses a different set of molecular markers. *Bmp2* and *Tgf*β*2* are expressed in the OFT, AVC and IFT since early looping stages and are important for further development of the non-chamber myocardium [7], [8]. Thus, differentiation of the primary myocardium and looping are the first steps of heart remodeling.

*Tbx5* plays a role during chamber formation despite being expressed throughout the extent of the heart tube. It has been shown that Tbx5 synergistically interacts with Nkx2.5 in the activation of the *ANF* regulatory element, responsible for expression of *Anf* in the cardiac chambers [9]. A similar regulatory element, composed of TBE and NKE binding sites, has been found in the promoters of other chamber-specific genes, such as *Cx40* and *Cx43* [10]. The lack of expression of chamber specific genes in non-chamber myocardium has been attributed to the expression of *Tbx2* which is mutually exclusive with that of chamber myocardium markers [11]. Tbx2 cooperates with Nkx2.5 and represses transcription from *ANF* regulatory element, serving as inhibitors of the chamber-specific genetic program. Supporting this model, misexpression of *Tbx2* under the control of β*-MHC* promoter results in the lack of chamber growth and downregulation of *Anf, Chisel* and *Cx40* [12]. However, mice with targeted mutations in *Anf*, *Chisel* or *Cx40* have normal heart development and reach adulthood [13]–[16], suggesting that the absence of their expression in β*-MHC::Tbx2* mice does not account for the lack of chamber growth. Recently, it has been shown that Tbx2 represses transcription of the cell cycle gene *Nmyc1*. The ectopic expression of *Tbx2* throughout the heart tube has been suggested to cause the hypoplastic phenotype observed in *Tbx20* mutants [17]–[19]. The chamber-specific genetic program is not initiated in *Tbx20* mutants, suggesting that downregulation of *Tbx2* in the chamber myocardium is crucial for normal heart development. Although *Nmyc1* mutant hearts are hypoplastic [20], [21], the cardiac chambers are formed and the overall phenotype is much less severe than that of *Tbx20* mutant or β*-MHC::Tbx2* hearts, suggesting that regulation of additional downstream targets of Tbx2 may be involved in chamber and non-chamber myocardium differentiation. Moreover, ablation of *Tbx2* causes defects in the AVC and a failure in OFT septation in only a quarter of the homozygous embryos [22], indicating that other factors are able to compensate for the lack of Tbx2.

*Tbx3* is co-expressed with *Tbx2* in the heart in looping stages and biochemical studies suggest Tbx3, like Tbx2, is capable of repressing transcription from the *ANF* element in chamber specific genes [10]. In humans, haploinsufficiency of *TBX3* causes the Ulnar Mammary syndrome (UMS), a pleiotropic disorder that typically presents defects in limb, mammary gland, tooth, hair and apocrine gland development [23]. This autosomal dominant disorder is fully penetrant, however there is a large variability in the clinical presentation among affected individuals and cardiac anomalies have recently been reported [24], [25]. Mice lacking *Tbx3* are not viable and display a wide range of onset of lethality, between E10.5 and E16.5, which correlates with the variability of clinical presentation in UMS patients [26]. Although *Tbx3* is expressed in restricted domains in the mouse and human hearts from early stages [10], heart formation has not been specifically addressed in *Tbx3* mutant embryos.

The processes of heart looping and chamber formation are less well studied in other vertebrates. In zebrafish, it is known that the initial broad expression of *bmp4* and *versican* become restricted to the myocardium at the level of the AVC during initiation of looping [27], [28]. As for chamber markers, zebrafish *anf* is expressed in chamber myocardium after looping is well under way [29]. However, the mechanism whereby heart remodeling occurs in zebrafish remains to be elucidated.

The simplicity and accessibility of the two-chambered heart in zebrafish allow a deeper analysis of the complex morphogenetic events involved in heart development. In this report, we show that novel *Tbx2* and *Tbx3* zebrafish homologs prevent initiation of the chamber-specific genetic program and growth in non-chamber myocardium. We also analyze cardiac defects in mice *Tbx3* mutant embryos and show that Tbx3 plays a role in heart remodeling. Together our findings reveal the importance of a tight equilibrium between cell proliferation and differentiation in order to achieve proper organogenesis.

## Results

### Zebrafish *tbx3b* and *tbx2a* are required for heart looping and AVC formation

**Figure 1:**
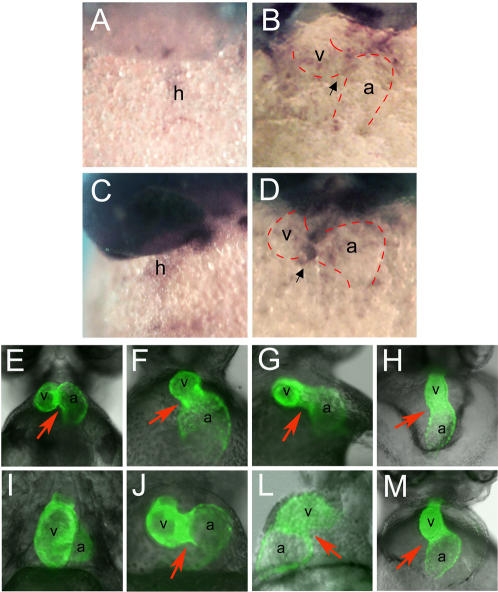
Aberrant morphology of the AVC in the absence of zebrafish *tbx3b* and *tbx2a*. (A–D) Whole mount RNA *in situ* hybridization of zebrafish *tbx3b* and *tbx2a* expression; ventral views show the heart in its maximum extension from the anterior to the posterior pole. Zebrafish *tbx3b* is expressed throughout the extent of the heart tube at 31 hpf (A) and becomes restricted to the AVC at 42 hpf (B). Zebrafish *tbx2a* is expressed al low levels throughout the extent of the linear heart tube at 31 hpf (C). At 42 hpf *tbx2a* transcripts are present in the AVC at high levels (D). Black arrow points to the AVC. (E–M) Ventral views of the heart of a *mlc2a::GFP* transgenic line that expresses GFP in the myocardium, at 48 hpf (E–H) and 72 hpf (I–M). (E) In wild type 48 hpf embryos, the atrium has moved upward and is positioned at the same anterior-posterior level as the ventricle; (I) later the atrium becomes localized dorsal to the ventricle at 72 hpf. (F, J) Injection of 2.5 ng of *tbx3b* morpholino into one-cell stage embryos results in delayed heart looping and abnormal AVC. (G, L) Injection of 10 ng of *tbx2a* MO results in a similar delay in heart looping and an enlargement of the AVC. (H, M) Injection of both *tbx3b* and *tbx2a* MOs, at 1.75 and 5 ng respectively, results in the failure to form the AVC constriction and absence of looping. Red arrow indicates the AVC. a, atrium; h, heart; v, ventricle.

We isolated two novel Tbx genes in zebrafish, which belong to the *Tbx2* subfamily. Based on sequence similarity and expression patterns we named them *tbx3b* and *tbx2a* (Fig. S1). *tbx3b* encodes a putative protein of 694 amino acid residues with overall similarity to human TBX3 of 67.8% and to mouse Tbx3 protein of 67.3%. *tbx2a* encodes a putative protein of 687 amino acid residues with 61.8% similarity to mouse Tbx2, 63.1% to human TBX2 and 78.2% to zebrafish tbx2b. During heart development, *tbx3b* is first expressed throughout the extent of the heart tube and becomes restricted to the AVC and OFT at 33 hpf (Fig. 1A,B). Zebrafish *tbx2a* is first weakly expressed throughout the extent of the heart tube becoming later restricted to the AVC (Fig. 1C,D). Thus, the domain of expression of *tbx3b* and *tbx2a* in the heart is similar to that of the mouse counterparts [10], [22].

The expression pattern of *tbx3b* and *tbx2a* suggested that they might play a role in heart regionalization in zebrafish. Morpholino oligonucleotides (MO) were used to knock down *tbx3b* and *tbx2a* in a transgenic line expressing GFP in the heart, *mlc2a::GFP* [30]. The efficacy of the MOs to inhibit translation of *tbx3b* and *tbx2a* mRNA were confirmed by co-injecting *tbx3b:EGFP* or *tbx2a:EGFP* fusion constructs and examining fluorescent signal (Fig. S2). Injection of *tbx3b* MO resulted in a growth delay from 36 hpf onwards, absence or reduction of fin buds and misformation of the otic vesicle and lethality past 3 dpf (I.R. and J.C.I.B., unpublished observations). In control embryos at 48 hpf the AVC was a tight constriction between the ventricle and the atrium and these two chambers were at the same anterior-posterior level (Fig. 1E). In stage-matched *tbx3b* morphant embryos, the atrium-ventricle border was wide and looping was delayed (Fig. 1F). By 3 dpf, the pericardiac cavity was severely swollen, looping was incomplete and the presumptive AVC remained wide (Fig. 1J). Loss of function of *tbx2a* resulted in similar heart defects, with incomplete looping and lack of AVC constriction (Fig. 1G,L). However, in contrast to *tbx3b* morphants, the fin buds and the otic placodes developed normally in *tbx2a* morphants and there was no growth delay (I.R. and J.C.I.B., unpublished observations), suggesting that these genes play specific and non-overlapping roles in the development of other organs. Upon co-injection of both *tbx3* and *tbx2a* MOs we observed that fin bud was absent and the otic placode was reduced (Fig. S2). In the heart of double morphants, the AVC failed to form, and there was no indication of looping (Fig. 1H,M). Although development of other organs was not more affected in double morphants than in *tbx3b* MO-injected embryos, the heart phenotype was more severe when both *tbx3b* and *tbx2a* were knocked down, implying that tbx3b and tbx2a play non-redundant roles in cardiac development.

**Figure 2:**
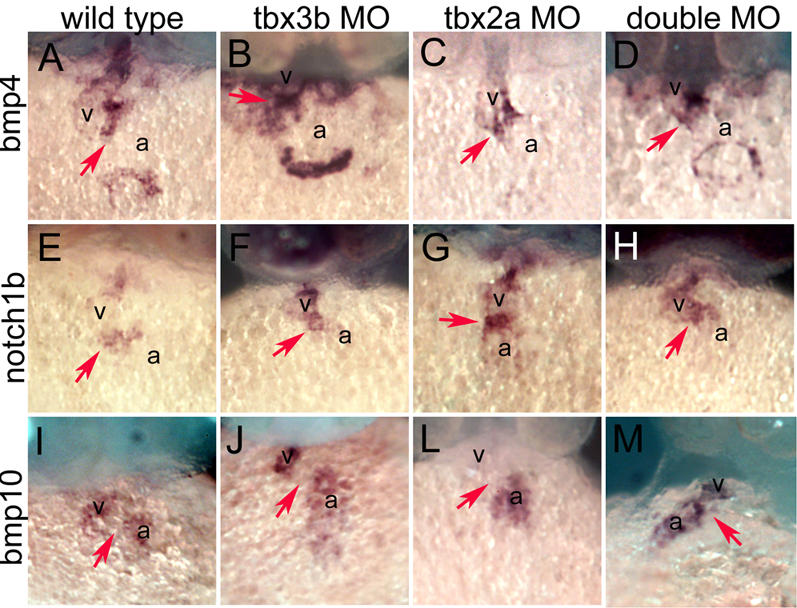
*in situ* hybridization analysis of cardiogenesis in the absence of *tbx3b* and *tbx2a*. Ventral views of the heart in its maximum extension from the anterior to the posterior pole. (A) *bmp4* is expressed in the non-chamber myocardium of the heart, the OFT, the AVC and the IFT. (B, C, D) The expression pattern of *bmp4* is normal in 42 hpf *tbx3b*, *tbx2a* and double morphants. (E) *notch1b* is expressed in the whole endocardium of the heart tube, but becomes restricted to the endocardium at the level of the OFT and AVC upon looping. (F, G, H) Expression of *notch1b* is maintained in the endocardium at the level of the chamber myocardium in 48 hpf *tbx3b*, *tbx2a* and double morphants, in contrast to wild type. (I) *bmp10* is expressed exclusively in chamber myocardium at 42 hpf (J, L, M) In 42 hpf *tbx3b* and *tbx2a* morphants *bmp10* is expressed in the chamber myocardium and is downregulated in the AVC, as is the case in wild type, but it is not downregulated from the AVC in double morphants. Red arrow indicates the AVC. a, atrium; h, heart; v, ventricle.

To test if the absence of the AVC constriction and looping were convoyed by defects in the heart regionalization, we assessed the expression of AVC markers in zebrafish. The expression domains of *bmp4* and *bmp2b* in OFT, AVC and IFT of the heart were not altered in single and double morphant embryos (Fig. 2A–D, data not shown). The expression domain of *notch1b*, which normally becomes restricted to the endocardium at the level of the AVC and OFT at 45 hpf [31], was expanded into the ventricle endocardium of *tbx3b* and *tbx2a* morphant embryos at 48 hpf (Fig. 2E,F,G), as well as in stage-matched double morphant embryos (Fig. 2H). Zebrafish *anf* is first expressed throughout the primitive heart tube and later becomes expressed exclusively in the chambers (our unpublished observations) [29]. In *tbx3b*, *tbx2a*, and double morphants, however, *anf* transcripts continued to be expressed throughout the extent of the cardiac myocardium at 48 hpf (data not shown). Zebrafish *bmp10* is expressed in the heart in a dynamic pattern reminiscent of zebrafish *anf* as well as in the otic vesicle and ventricle of the brain (Fig. S3). In *tbx3b* and *tbx2a* morphant embryos, *bmp10* was downregulated from the AVC as in control embryos (Fig. 2I–L). However, in double morphants *bmp10* was present throughout the extent of the heart tube (Fig. 2M). Together these results show that the initial specification of chamber and non-chamber myocardium occurs in single and double morphants, but further development is impaired.

### Ectopic presence of Tbx3 and Tbx2 results in the absence of chamber growth

**Figure 3:**
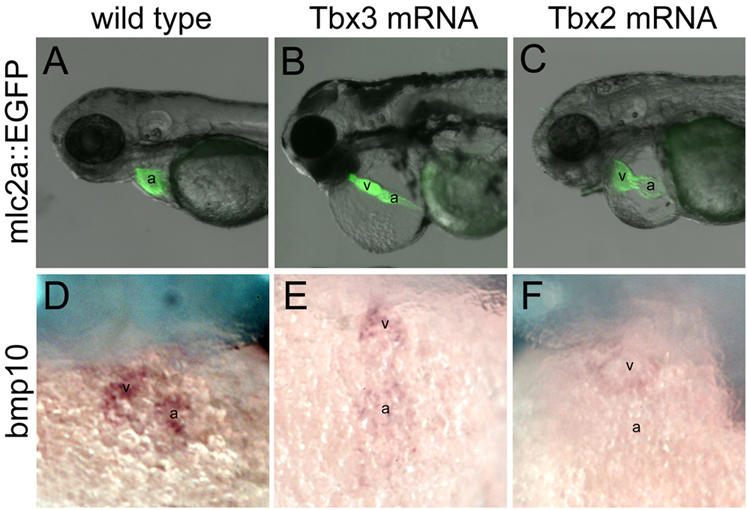
Heart morphology in embryos overexpressing Tbx3 and Tbx2. Side views (A–C) and ventral views (D–F) of wild type embryos (A, D) and embryos injected with 100 pg capped mRNA for mouse Tbx3 (B, E) or human TBX2 (C, F). (A, B, C) *mlc2a::GFP* embryos at 60 hpf; (D, E, F) 48 hpf. (B, C) 44.5% of Tbx3-injected embryos and 60% of Tbx2-injected embryos present a pipe-like heart that lacks chamber growth and looping. (D, E, F) Zebrafish *bmp10* is strongly expressed in the chamber myocardium in wild type embryos, but is significantly downregulated from the heart in Tbx3- or Tbx2-injected embryos. a, atrium; v, ventricle.

We next asked if the ectopic expression of Tbx3 and Tbx2 in chamber myocardium was able to prevent chamber formation. Tbx3-injected embryos developed at comparable rates as the GFP-injected embryos. At 60 hpf, 44.5% of the embryos overexpressing Tbx3 presented a swollen pericardiac cavity and a delay in heart looping. Most notably, however, was the lack of chamber growth, which, added to the increasing pericardial swelling due to low circulation, gave rise to a thin heart tube that stretched from the base of the head to the yolk (Fig. 3B; compare to 3A), a condition previously described as pipe-like heart [27]. In 60% Tbx2-injected embryos the pericardiac cavity was swollen from 48 hpf onwards (Fig. 3C). A similar pipe-like heart phenotype was visible at 3 dpf. In line with the lack of chamber growth, zebrafish *bmp10* was significantly downregulated in the heart in Tbx3- and Tbx2-injected embryos (Fig. 3D,E,F). Transcripts of *anf* were not downregulated from the AVC of Tbx3- and Tbx2-overexpressing hearts at 48 hpf and presented levels of expression similar to the wild type 24 hpf heart (data not shown). The overexpression of Tbx3 or Tbx2 did not affect the expression of *notch1b* or *bmp4* (data not shown). Thus, the specification and differentiation of non-chamber myocardium is not altered in the presence of ectopic Tbx3 or Tbx2 in the heart tube. However, the presumptive chambers do not grow from the primitive heart tube, and maintain levels of expression of chamber markers characteristic of primary myocardium.

### *tbx3b* and *tbx2a* are required to establish the pattern of proliferation during heart looping

**Figure 4:**
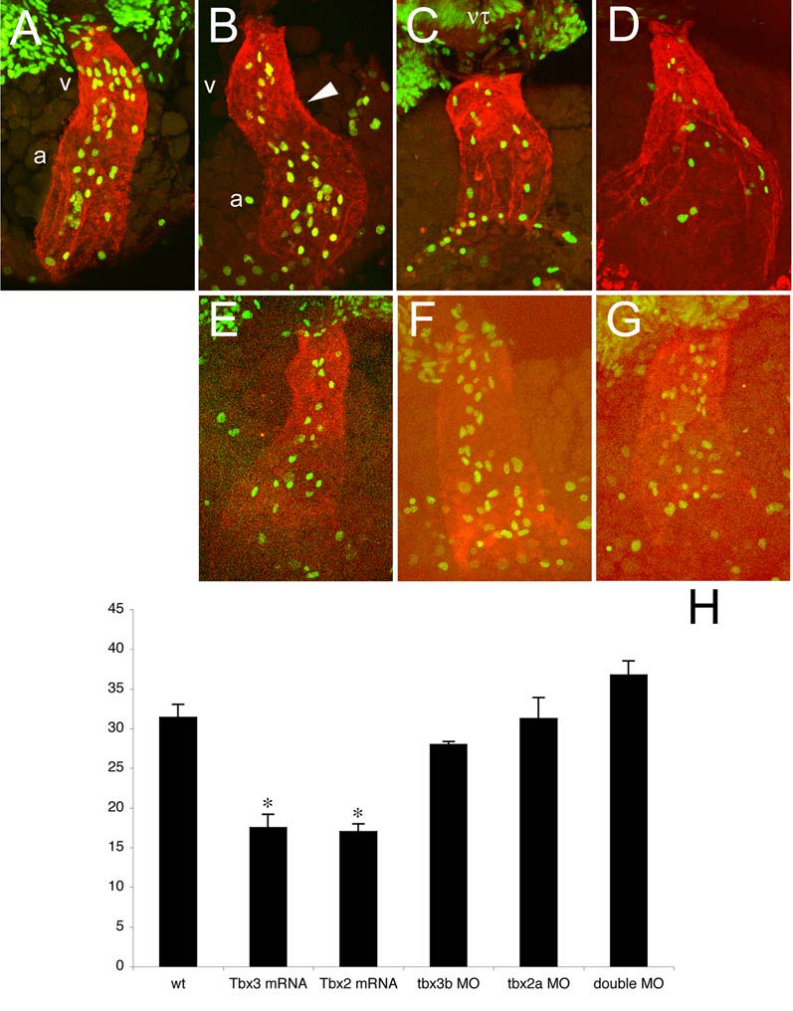
The pattern of proliferation along the heart tube is dynamic and is regulated by Tbx3 and Tbx2. All images represent reconstructions of confocal Z-stack sections imaged on whole embryos at 31hpf (A) and 33 hpf (B–G). (A, B) During the first steps of looping, the pattern of proliferation shifts from homogenous throughout the heart tube (31 hpf, A) to a heterogenous one in which dividing cells are more concentrated in the future chambers (33 hpf, B). (C, D) In Tbx3- (C) and Tbx2- (D) injected embryos at 33 hpf this shift has not occurred and the number of dividing cells was significantly decreased and dividing cells were homogenously distributed (H). (E–G) MO-injected embryos against *tbx3b* (E), *tbx2a* (F) or both (G) display the same (E, F) or higher (G) number of proliferating cells than wild type at 33 hpf. However, proliferating cells remain homogenously distributed throughout the heart tube. (H) Histogram showing the average of the total number of BrdU positive cells in the heart of 33 hpf embryos: wt, 31.4±1.661 (n = 5); Tbx3 mRNA, 17.5±1.708 (p<0.001; n = 6); Tbx2 mRNA, 17.0±1.000 (p<0.001; n = 7); *tbx3b* MO, 28.0±0.408 (n = 4); tbx2a MO, 31.3±2.658 (n = 4); double MO, 36.8±1.797 (n = 4). a, atrium; h, heart; nt, neural tube; v, ventricle.

The absence of chamber growth in Tbx3- and Tbx2-injected embryos prompted us to investigate the cell division and apoptosis levels during heart looping. In wild type embryos between 31 and 48 hpf, no significant numbers of apoptotic cells, as evaluated by acridine orange staining, were observed in the heart (data not shown). Additionally, no differences were observed between Tbx3- or Tbx2-injected and wild type embryos in the number of apoptotic cells in the heart tube (data not shown). To study the pattern of proliferation in the looping heart we gave a one-hour pulse of BrdU by injecting a BrdU solution in the pericardiac cavity. In 31 hpf wild type embryos the heart presented no signs of looping and chamber formation. The BrdU-positive cells were equally distributed throughout the extent of the heart tube (Fig. 4A). At 33 hpf, the anterior region of the heart tube, the future ventricle, has jogged to the right, allowing a rough distinction between the two future chambers and the future AVC. At this stage, the BrdU positive cells were more concentrated in the future chamber regions, leaving the AVC region devoid of BrdU-positive cells (Fig. 4B). At 36 hpf the initial phase of heart looping is already completed and it is possible to clearly identify the ventricle and the atrium, as well as the OFT and the AVC. Thus, a dynamic pattern of cell proliferation along the heart tube accompanies chamber outgrowth, and a crucial differentiation step occurs at 33 hpf, concomitant with the rightward jogging of the future ventricle.

The pre-looping heart tube of Tbx3-injected embryos appears normal in size and morphology (data not shown). However, at 33 hpf there were no signs of looping and the dividing cells were homogenously distributed throughout the heart tube, with no obvious separation between ventricle and atrium (Fig. 4C). Moreover, the total number of BrdU-positive cells in the heart was significantly lower when compared to wild type embryos (Fig. 4H). Similar altered numbers and distribution of BrdU-positive cells were observed in the hearts in Tbx2-injected embryos at 33 hpf (Fig. 4D,H). These results indicate that the lack of chamber outgrowth in the presence of ectopic Tbx3 and Tbx2 is due to a decrease in the cell proliferation levels in the future chamber myocardium. In 33 hpf stage-matched *tbx3b* or *tbx2a* morphants, the distribution of dividing cells was not regionalized as in control hearts (Fig. 4E,F,G). The 33 hpf heart tube of double morphant embryos displayed an homogenous distribution of BrdU-positive cells and the total number of dividing cells was slightly higher than in wild type (Fig. 4H). Differences in the number of BrdU-positive cells in the AVC myocardium may be difficult to detect due to the small number of cells in this region. Together these results demonstrate that *tbx3b* and *tbx2a* are crucial for the establishment of the dynamic pattern of cell proliferation in the heart tube and further show that perturbation of this pattern may have drastic consequences for heart looping.

### Ablation of Tbx3 in mice causes cardiovascular defects

In order to investigate if the role of Tbx3 in zebrafish heart morphogenesis is conserved in mammals, we analyzed the cardiovascular development in mice embryos lacking Tbx3 function. Mice with a targeted mutation for *Tbx3* were generated (Materials and Methods, Fig. S4). Homozygous *Tbx3* null embryos presented poorly developed yolk sac vasculature, truncated hindlimbs and were smaller than their littermates, similar to what has been described for another *Tbx3* mutant [26].

**Figure 5:**
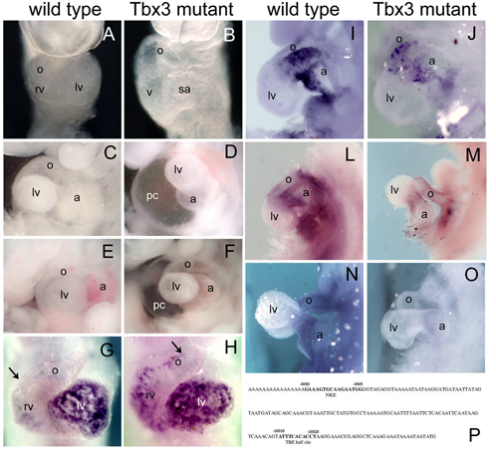
Mice with a targeted mutation for *Tbx3*, *Tbx3^neo^* display cardiac defects. (A, B) Severely affected *Tbx3* mutant embryos (B) present a delay in heart looping at E9.5, in which the ventricle is still at the same dorsoventral position as the atrium. (C, D) At E10.5, the most affected *Tbx3* mutant embryos display obvious heart defects, including lack of the constriction in the AVC and absence of looping in a swollen pericardiac cavity (D), compared to wild type (C). (E, F) E11.5 *Tbx3* null homozygous embryos present a significant delay in heart looping and pericardiac swelling compared to wild type. (G–O) Whole mount *in situ* hybridization analysis at E9.5; ventral views (G, H) and lateral views (I–O). (G, H) Upon looping initiation, *Bmp10* starts to be expressed in the chamber myocardium. However, *Bmp10* was ectopically expressed in the non-chamber myocardium of the heart of *Tbx3* mutant embryos (arrow in G, H). (I, J) *Bmp2* is expressed in the AVC myocardium at E9.5 and was not altered in *Tbx3* mutant embryos. (L, M) *TGF*β*2* is normally expressed in the non-chamber myocardium of the looping heart. In *Tbx3* mutant embryos, expression of *TGF*β*2* is downregulated in the heart. (N, O) *Smad6* is expressed in the endocardium at the level of the OFT and AVC at E9,5. However, in *Tbx3* mutant embryos, expression of *Smad6* is absent. (P) Consecutive NKE and TBE binding sites are found in the human *BMP10* promoter, between 9800 and 10100 bp upstream the ATG codon. a, atrium; lv, left ventricle; o, outflow tract; pc, pericardiac cavity; rv, right ventricle; sa, sinoatrial region.

In *Tbx3* mutant embryos, we observed a large phenotypic variability in the time of onset and degree of the heart defects. In 4/40 homozygous embryos collected 9.5 days post coitum (E9.5), a delay in heart looping was evident (Fig. 5A,B). In 33% of the *Tbx3* mutant embryos collected at E10.5 (n = 21), the pericardiac cavity was swollen and the heart incompletely looped (Fig. 5C,D). The defects in looping ranged from a mild delay to a completely unbent tube (Fig. 5D). The constriction at the AVC was also enlarged when compared to wild type or heterozygous littermates. At later stages, all *Tbx3* mutant embryos were readily distinguishable from their littermates in that they were smaller and presented a reduced hindlimb bud (our unpublished observations). 43% *Tbx3* mutant embryos recovered at E11.5 (n = 7) presented a swollen pericardial cavity with an unlooped heart (Fig. 5E,F). These observations suggest that there is a great phenotypic variation in the severity of the cardiovascular defects in *Tbx3* mutant embryos. Nevertheless, the most severe mutants display cardiac anomalies comparable to the heart defects seen in *tbx3b* morphant embryos.

To test if the AVC is correctly specified in *Tbx3*-mutant embryos, we analyzed the expression pattern of *Bmp2*, which is expressed in the AVC and is crucial for the formation of atrioventricular valves in the mouse [8]. *Bmp2* transcripts were present in the AVC of *Tbx3* mutant embryos at E9.5 (Fig. 5I,J), suggesting that this non-chamber region is properly specified. To test if further development of the AVC was occurring normally, we analyzed *TGF*β*2* and *Smad6* which are expressed in the OFT and the AVC at the level of the myocardium and endocardium, respectively, and are required for cushion mesenchyme formation [32], [33]. In *Tbx3* null mutant embryos, both *TGF*β*2* and *Smad6* were downregulated in the non-chamber myocardium (Fig. 5L–O). Expression of *Msx2*, another marker of AVC myocardium, was downregulated in *Tbx3* mutant embryos at E9.5 (data not shown). Thus, the early specification of the AVC seems to occur normally, however further differentiation of this myocardium is perturbed in *Tbx3* null mutants.

Next we analyzed the expression of chamber myocardium markers, *Anf* and *Bmp10*. In the most severe class of *Tbx3*-null mutants, *Anf* was upregulated in the region between the ventricle and the atrium (data not shown). *Bmp10* is normally expressed in the chambers at E9.5 marking the future trabeculating myocardium [6]. In *Tbx3*-null homozygous embryos *Bmp10* transcripts were found ectopically in the OFT and the AVC at E9.5 (Fig. 5G,H), suggesting that in the absence of Tbx3, *Bmp10* is activated in non-chamber myocardium. We found NKE- and TBE-sites in the promoter region of human *BMP10* (Fig. 5P), which suggest that *Bmp10* is subject to similar transcriptional regulation as *Anf* and *Cx40* [4]. Tbx5 is required for chamber formation [34] and was normally expressed in *Tbx3* mutant embryos (data not shown). The analysis of the expression patterns of chamber and non-chamber markers in *Tbx3* mutant embryos advocates that the initial specification of non-chamber myocardium occurs normally in these embryos. In spite of this, further development of the AVC is not achieved and instead, chamber markers are upregulated. Moreover, the most severe class of *Tbx3*-null mutant embryos displays heart defects that are similar to zebrafish *tbx3b* morphants with respect to morphology and the degree of development, indicating that the mechanisms of AVC formation are conserved among vertebrates.

**Figure 6:**
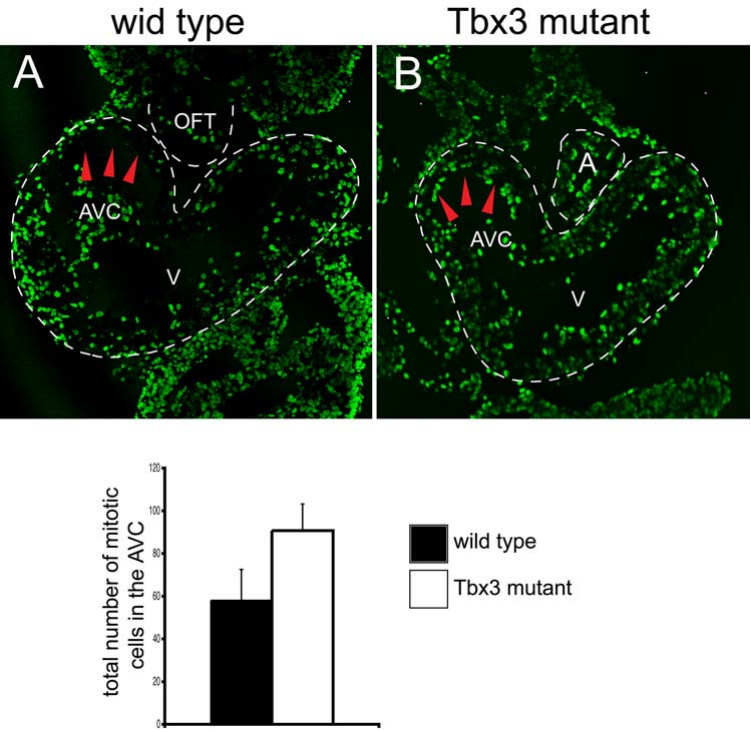
The pattern of proliferation in the E9.5 heart tube is altered in *Tbx3* mutant hearts. Immunohistochemical analysis of cell proliferation in E9.5 wild type (A) and *Tbx3* mutant (B) hearts using antibodies against BrdU (A, B) or phospho-Histone 3 (C). (A, B) Representative sections at the level of the AVC of wild type and mutant hearts are shown. The heart is outlined by a discontinuous white line. The density of BrdU positive cells is higher in the AVC of mutant hearts than in the AVC of wild type hearts (red arrowheads). (C) The number of phospho-Histone3-positive cells in the AVC was counted in consecutive heart sections at the level of the AVC of four wild type embryos with 30–31 somites and three mutant embryos with 30–32 somites. The average number of mitotic cells for the AVC is represented in a histogram. The number of mitotic cells in the AVC is significantly increased (P<0.010) in mutant embryos (90.7±12.7), compared to wild type embryos (58.3±14.5). A, atrium; AVC, atrioventricular canal; OFT, outflow tract; V, ventricle.

Previous studies show that the sinoatrial region, the AVC and, to a lesser extent, the OFT present lower levels of cell proliferation compared to the chamber myocardium [35], [36]. Since the AVC constriction is unusually wide in *Tbx3*-null mutants and there is ectopic activation of the chamber genetic program, we analyzed whether the pattern of cell proliferation was altered in these mutants. Consistent with previous findings [35], wild type E9.5 embryos labeled with a one-hour pulse of BrdU present a lower density of BrdU-positive cells in the OFT and the AVC when compared to the ventricle or the common atrium (Fig. 6A, data not shown). *Tbx3* mutant embryos subjected to the same treatment displayed densities of proliferating cells in the OFT similar to wild type embryos. However, the density of proliferating cells at the level of the AVC myocardium was comparable to that of the ventricle myocardium, rather than to that of the wild type AVC myocardium (Fig. 6A,B and data not shown). In addition to the analysis of BrdU-labeled cells, we also examined the pattern of mitotic cells in the looping heart by detection of phospho-Histone3-positive cells. Exhaustive counting of the phospho-Histone3-positive cells in each section revealed that proliferation levels were not altered in the OFT, the ventricles or the common atrium at E9.5 in *Tbx3* mutant embryos (n = 2/3) relative to wild type counterparts (n = 3/4; data not shown). In spite of this, the number of dividing cells was significantly increased in the AVC of *Tbx3* mutant embryos (n = 3/3) when compared to wild type embryos (4/4; Fig. 6C). These results suggest that Tbx3 is an important regulator of the rate of proliferation in the AVC and reveal that the role of Tbx3 is conserved in zebrafish and mouse heart development.

## Discussion

In this report, we have studied the role of two novel zebrafish genes, *tbx3b* and *tbx2a*, in the process of heart remodeling and the associated differential cell proliferation. Incomplete looping was observed both after gain- and loss-of-function experiments. The pattern of cell proliferation along the heart tube was perturbed in either case, suggesting that differential rates of cell proliferation are important for proper looping to occur. In the absence of *tbx3b* and *tbx2a*, the broadening of the AVC constriction may hamper heart tube bending by abolishing the flexion point. On the other hand, the chamber growth out of the primary heart tube may be a permissive factor for the bending of the heart tube along with other intrinsic and extrinsic biomechanical driving forces [37], as evidenced by the absence of looping in the gain-of-function experiments. Supporting this hypothesis, *heartstrings* mutant and *hrT* morphants display an absence of chamber formation and impaired heart looping [27], [38]. Perturbation of heart looping in *fog1* morphants does not correlate with lack of chamber growth [39], suggesting that chamber growth is not sufficient for proper looping to occur. In mice, lack of Tbx5 and Nkx2.5 results in an hypoplastic heart and absence of looping [11], [34], indicating that lack of looping in the absence of chamber growth is conserved in mice. Tbx3 and Tbx2 may also be involved in other aspects of the process of heart looping not related to chamber growth. It has been previously shown that Tbx genes can regulate extracellular matrix composition and cell adhesion [40], which are important factors in the bending of the heart tube [41].

Frequently during embryonic development, two adjacent tissues present disparate rates of cell division and the maintenance of these discrepancies is important for proper morphogenesis to occur. In the heart, the rate of cell proliferation is higher in chamber myocardium compared to the non-chamber myocardium in mouse [35], [36] and there is a correlation between absence of chamber formation and lower levels of cell proliferation [42], [43]. Analysis of the pattern of cell proliferation along the extent of the zebrafish heart tube revealed that cell proliferation is dynamic throughout heart development, increasing upon initiation of chamber formation (this study). We show that tbx3b and tbx2a are necessary to break the uniform distribution of dividing cells in the zebrafish heart by repressing cell proliferation. T-box transcription factors have been previously implicated in the control of cell proliferation in several developmental contexts [19], [44], [45] and in one case, this control has been shown to be direct: Tbx2 downregulates expression of *Nmyc1* in the context of heart development [43]. Thus, one possible mechanism for cell proliferation control in the non-chamber myocardium by tbx2a and tbx3b is by regulation of expression of cell cycle genes.

Concomitant with differential increase in cell proliferation in the heart tube is the activation of a chamber-specific genetic program in chamber myocardium. tbx2a and tbx3b repress the transcription of *bmp10* in the AVC. Mouse Bmp10 has been shown to be required for chamber growth and maturation [42], indicating that *Bmp10* is a chamber-specific gene that is actually required for chamber growth and maturation. In *Bmp10*-null hearts, the cell cycle inhibitor p57 (kip2) is upregulated and the rate of cell proliferation is lower than in wild type hearts [42]. The presence of putative NKE- and TBE-binding sites in tandem in upstream sequences of human *BMP10* suggest that regulation of this chamber marker could be similar to the that of *Anf*, *Cx40* and *Cx43* [4]. Thus, regulation of chamber versus non-chamber genetic programs may be achieved by Bmp10, which we show is altered in the absence of *tbx2a* and *tbx3b* in zebrafish and *Tbx3* in mouse.

Downregulation of *TGF*β*2* in the AVC myocardium was observed upon ablation of *Tbx3* in the mouse. It has been suggested that defects in cardiac looping are at the origin of cardiac defects of *TGF*β*2* mutant embryos, which display an overriding tricuspid valve and double outlet right ventricle [7]. Furthermore, TGFβ2 can trigger cell cycle arrest by induction of p21 expression in other contexts [46]. In the converse experiment, cardiac-restricted expression of a constitutively active form of the type I TGFβ receptor Alk5 results in defective heart looping, hypocellularity and upregulation of p21 [47]. These studies suggest that TGFβ2 must play role in heart remodeling and TGFβ2 downregulation in Tbx3-null hearts may explain the increase in cell proliferation and defect in heart looping observed.

Loss-of-function of tbx2a and tbx3b each independently resulted in malformation of AVC constriction due to altered cell proliferation rates and incomplete looping. Ablation of *Tbx2* or *Tbx3* independently in mouse also result in AVC malformation [22] and, in the case of *Tbx3*, in incomplete looping and altered cell proliferation rates in the AVC. These observations imply that the roles of Tbx2 and Tbx3 are conserved in both vertebrates. Upon knock down of both *tbx2a* and *tbx3b* we observed absence of AVC constriction and looping altogether and the defects in cell proliferation were more severe, suggesting that tbx2a and tbx3b play additive roles in heart morphogenesis. As repressors of transcription, tbx2 and tbx3 may simply have different transcriptional targets in the nucleus. Low similarity of protein sequences outside the T-box domain [48] may signify association with different cofactors, supporting this hypothesis. The identification of transcriptional targets and binding partners of Tbx2 and Tbx3 would clarify this issue.

Cardiac looping involves a complex set of movements of discrete regions of the heart tube that are correlated with the regionalization of the heart, i.e., the appearance of molecular and cellular characteristics specific for each heart region. A differential rate of cell proliferation along the heart tube results in growth of cardiac chambers out of the primary myocardium thereby creating the AVC constriction. Tbx2 and Tbx3 repress the induction of chamber-specific genes and cell proliferation in the AVC. The AVC constriction may act as a flexion point that facilitates the bending of the heart tube. On the other hand, defects in cardiac looping are at the origin of malformations in the valve s due to a failure in approaching the correct heart regions [7]. Thus, looping and regionalization are deeply interrelated processes that depend on each other to occur successfully. Our studies uncover a crucial role of Tbx3/2 transcription factors in the regulation of these processes and provide an entry-point for understanding the cellular bases that underlie heart tube looping and regionalization.

## Materials and Methods

### Zebrafish lines and Microinjection

Wild type zebrafish (AB) and the transgenic *mlc2a*::GFP line [30] were used in this study. pCS2+ vectors carrying cDNA inserts coding for mouse Tbx3, human TBX2 and green fluorescent protein (GFP) were synthesized using the SP6 mMessage Machine System according to the manufacturer's instructions (Ambion). Capped mRNAs and morpholino oligonucleotides (MO) were injected into one-cell stage embryos as described [49]. MOs were designed to specifically inhibit RNA translation of the targeted gene and were obtained from GeneTools. The sequences were as follows:

Control MO: 5′-CCTCTTACCTCAGTTACAATTTATA-3′ [50],

*tbx3b* MO: 5′-TGGATCTCTCATCGGGAAGTCCAG-3′,

*tbx2a* MO: 5′-ATCGGTGCATCCAAAAAGCCAGAT-3′.

Injection of the same amount of control MO did not produce any detectable defects.

### Cloning of zebrafish *tbx3b*, *tbx2a*, and *bmp10* genes

A zebrafish embryonic cDNA library was screened with low stringency hybridization using the T-box domain of zebrafish *tbx5* as a probe. 5′RACE-PCR was performed to obtain the open reading frame sequence for *tbx3b* and *tbx2a*. The sequences were submitted to NCBI molecular database. The zebrafish cDNA ensembl database (Zv5) was blasted with the cDNA sequence from mouse and human *Bmp10*. We obtained one cDNA sequence with significant similarity, which was amplified by RT-PCR. By comparing the expression pattern of this gene with the one from mouse *Bmp10* we concluded that it is zebrafish *bmp10*.

### Whole Mount *in situ* Hybridization

Whole-mount *in situ* hybridization for zebrafish embryos was performed as described [49]. The zebrafish antisense RNA probes for *nkx2.5*, *anf*, *bmp2a*, *bmp2b* and *has2* were obtained by RT-PCR of a cDNA fragment including the 3′ UTR, *bmp4*, *tbx5* and *versican* were previously described [49], [51] and *hrT* was kindly provided by D.Yelon. For mouse embryos, *in situ* hybridization was performed as described [49]. The mouse antisense probes for *Bmp10*, *Anf*, *Smad6*, and *TGF*β*2* were obtained by RT-PCR of a cDNA fragment including 3′ UTR.

### Analysis of cell proliferation

To analyze cell proliferation in the zebrafish heart, aprox. 2 nl of 1 mg/ml bromodeoxyuridine (BrdU) was injected in the pericardiac cavity of tricaine anesthetized embryos, which were fixed after one hour. The distribution of BrdU-positive cells along the progressive zone of the 48hpf fin bud confirmed that this approach is reliable (data not shown) [49]. Whole-mount immunofluorescence was performed using standard protocols using anti-BrdU antibody conjugated to fluorescein (Roche). BrdU-injected embryos were then stripped of their head, oriented in solidifying 0.5% agarose and photographed using a BioRad confocal microscope. To analyze cell proliferation in the mouse heart, pregnant female mice were IP injected with BrdU solution at 100 µg/g body weight. Embryos were recovered after 1 h, fixed and embedded in paraffin. Ten-micrometer serial sections were cut and processed for staining. Epitopes were recovered by boiling the slides for 20 minutes in unmasking solution (VectorLabs). Mice immunohistochemistry was performed according to standard protocols. The antibodies used were MF20, 1∶100, obtained from the Developmental Studies Hybridoma Bank, anti-BrdU conjugated to fluorescein, 1∶100 (Roche), anti-BrdU, 1∶200 (Accurate Chemical) and anti-phospho Histone3, 1∶300 (Ser10; Upstate).

### Generation of Tbx3 KO mice

Targeted disruption of the mouse *Tbx3* locus to produce the *Tbx3neo* allele by homologous recombination in embryonic stem (ES) cells was performed. Details of the targeting strategy and other analyses are shown in Supplemental Figure S4 and Supplemental Methods S1. In brief, a *loxP*-flanked *neo* selection cassette was inserted into the first exon of the *Tbx3* locus by homologous recombination in J1 ES cells. This *neo* cassette contains multiple stop codons that prevent translation of *Tbx3* mRNA into protein, thus we considered this generated allele as a null mutated allele, and called it *Tbx3neo*. Germ-line chimeras were generated by injection of targeted ES cells into C57BL/6 host blastocysts. Chimeras were mated with 129t females and the first progeny were confirmed to harbor the *Tbx3neo* allele by Southern analysis and PCR.

## Supporting Information

Figure S1 Zebrafis tbx3b and tbx2a are orthologues of mouse Tbx3 and Tbx2. Dorsal (A, C) and lateral views (B, D) of in situ hybridized 48 hpf embryos with tbx3b (A, B) and tbx2a (C, D). Asterisk indicates the fin bud. (E) Comparison of the amino acid sequence of the T-box domain of several Tbx2 subfamily proteins shows that tbx3b and tbx2a exhibit high similarity with other members from this family.
(3.00 MB TIF)

Figure S2 tbx3b and tbx2a efficiently prevent translation of their target messenger RNAs. (A–D) epiboly stage. Capped RNA encoding for tbx3b:EGFP or tbx2a:EGFP fusion proteins was injected with or without the respective MO to verify the efficiency of MOs to abolish translation of tbx3b and tbx2a mRNAs. In the presence of the MO, no fluorescent signal was present indicating that translation of the fusion constructs was abolished.
(1.74 MB TIF)

Figure S3 Expression pattern of zebrafish bmp10. Whole mount in situ hybridization of embryos at 24 hpf (A, B) and 60 hpf.(C, D). (A) dorsal view of the head; (B) lateral view; (C) lateral and dorsal view of the head and (D) ventral view of the heart. (A, B) Zebrafish bmp10 is expressed in the brain and the whole heart tube at 24 hpf. (C, D) At 60 hpf, bmp 10 is expressed in the myocardium of the heart chambers as well as in the otic vesicle. a, atrium; h, heart; op, otic vesicle; v, ventricle.
(3.24 MB TIF)

Figure S4 Targeting strategy to produce the Tbx3 null allele. (A) Schematic representation of genomic organization of the wild type mouse Tbx3 locus. (B) Diagram of the targeting strategy. Targeting construct showing the regions of homology and the site of insertion of the neo cassette in the Tbx3 locus. (C) Genomic southern analysis of EcoRV digested DNA of the ES cell clones.
(0.43 MB TIF)

Methods S1 (0.03 MB DOC)
