# Supplementary figures and images for: Tbx2 and Tbx3 Regulate the Dynamics of Cell Proliferation during Heart Remodeling
Source: PLoS One. 2007 Apr 25;2(4):e398. doi: 10.1371/journal.pone.0000398 (PMC1851989; doi:10.1371/journal.pone.0000398)

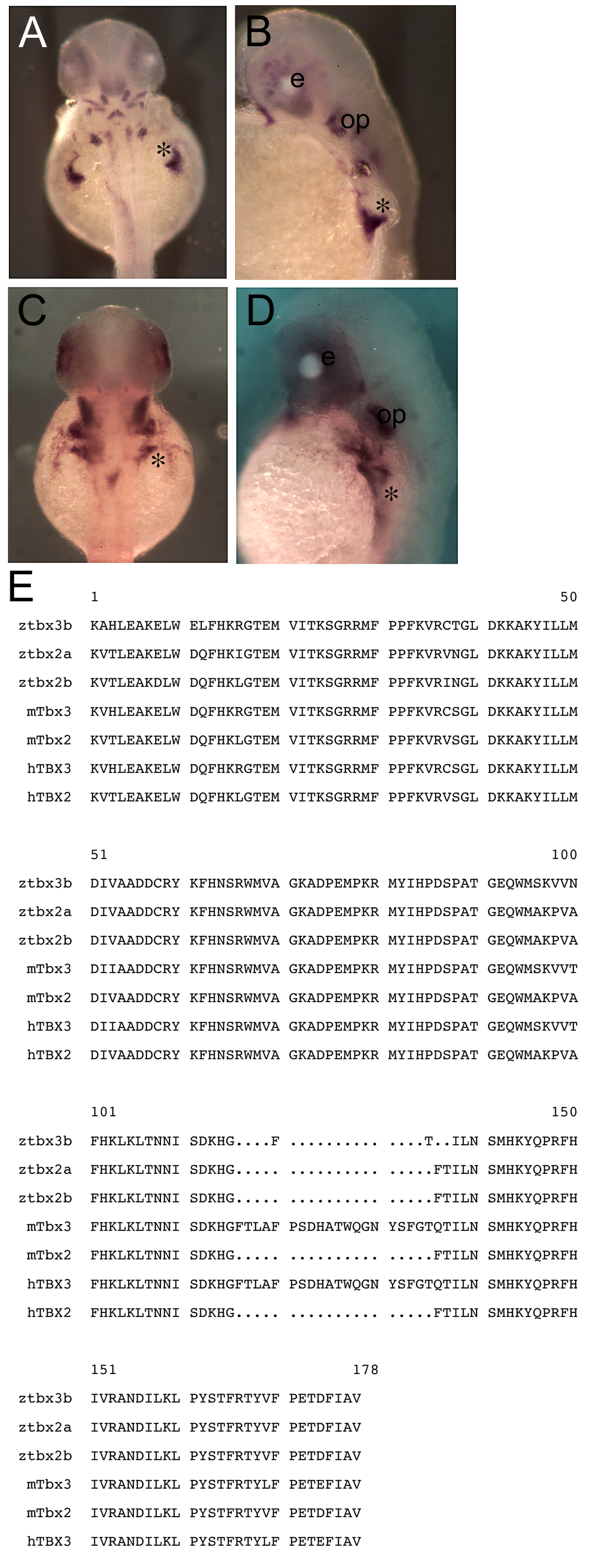

Supplement: Figure S1 — Zebrafis tbx3b and tbx2a are orthologues of mouse Tbx3 and Tbx2. Dorsal (A, C) and lateral views (B, D) of in situ hybridized 48 hpf embryos with tbx3b (A, B) and tbx2a (C, D). Asterisk indicates the fin bud. (E) Comparison of the amino acid sequence of the T-box domain of several Tbx2 subfamily proteins shows that tbx3b and tbx2a exhibit high similarity with other members from this family. (3.00 MB TIF) [file pone.0000398.s001.tif]

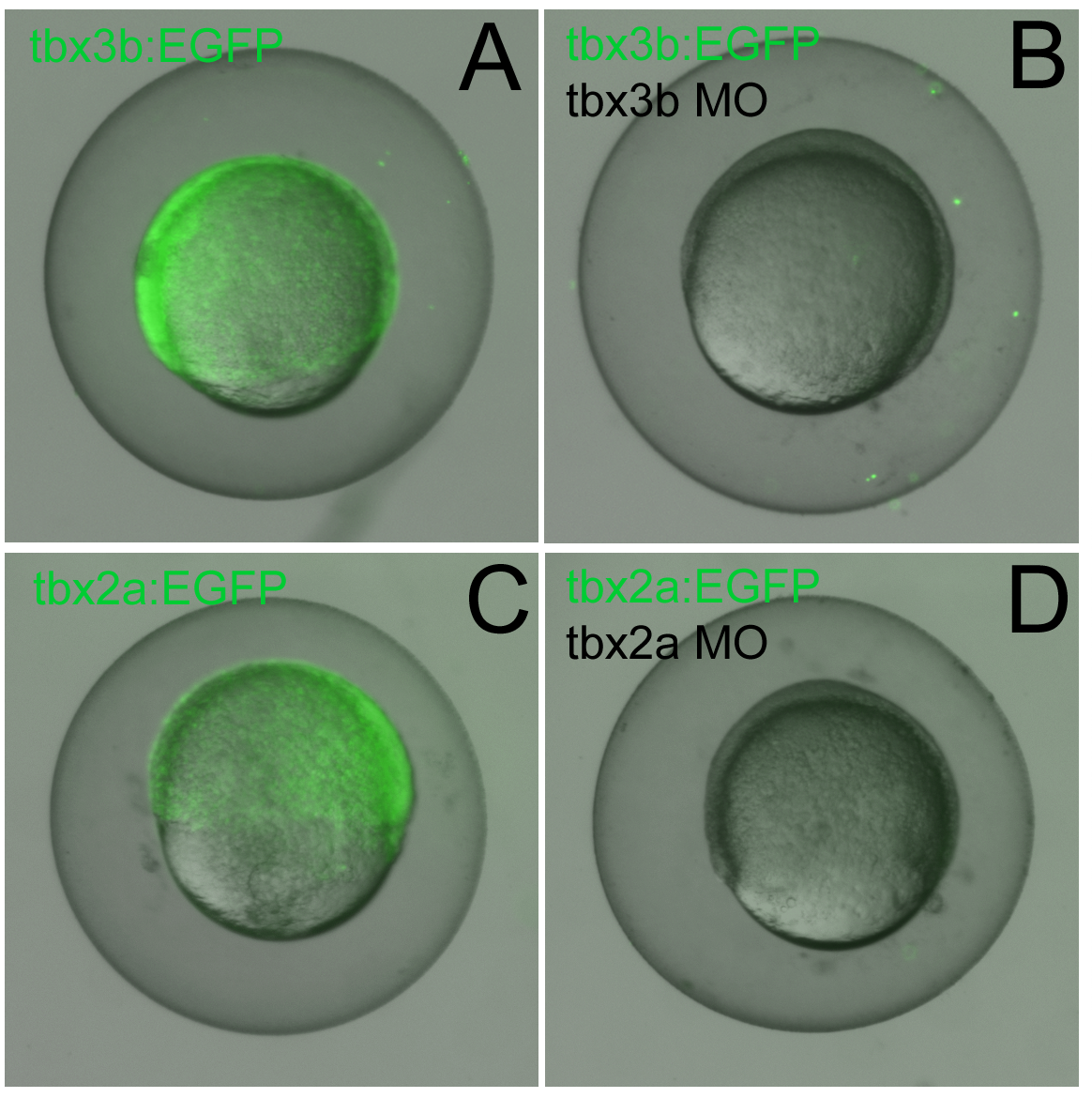

Supplement: Figure S2 — tbx3b and tbx2a efficiently prevent translation of their target messenger RNAs. (A–D) epiboly stage. Capped RNA encoding for tbx3b:EGFP or tbx2a:EGFP fusion proteins was injected with or without the respective MO to verify the efficiency of MOs to abolish translation of tbx3b and tbx2a mRNAs. In the presence of the MO, no fluorescent signal was present indicating that translation of the fusion constructs was abolished. (1.74 MB TIF) [file pone.0000398.s002.tif]

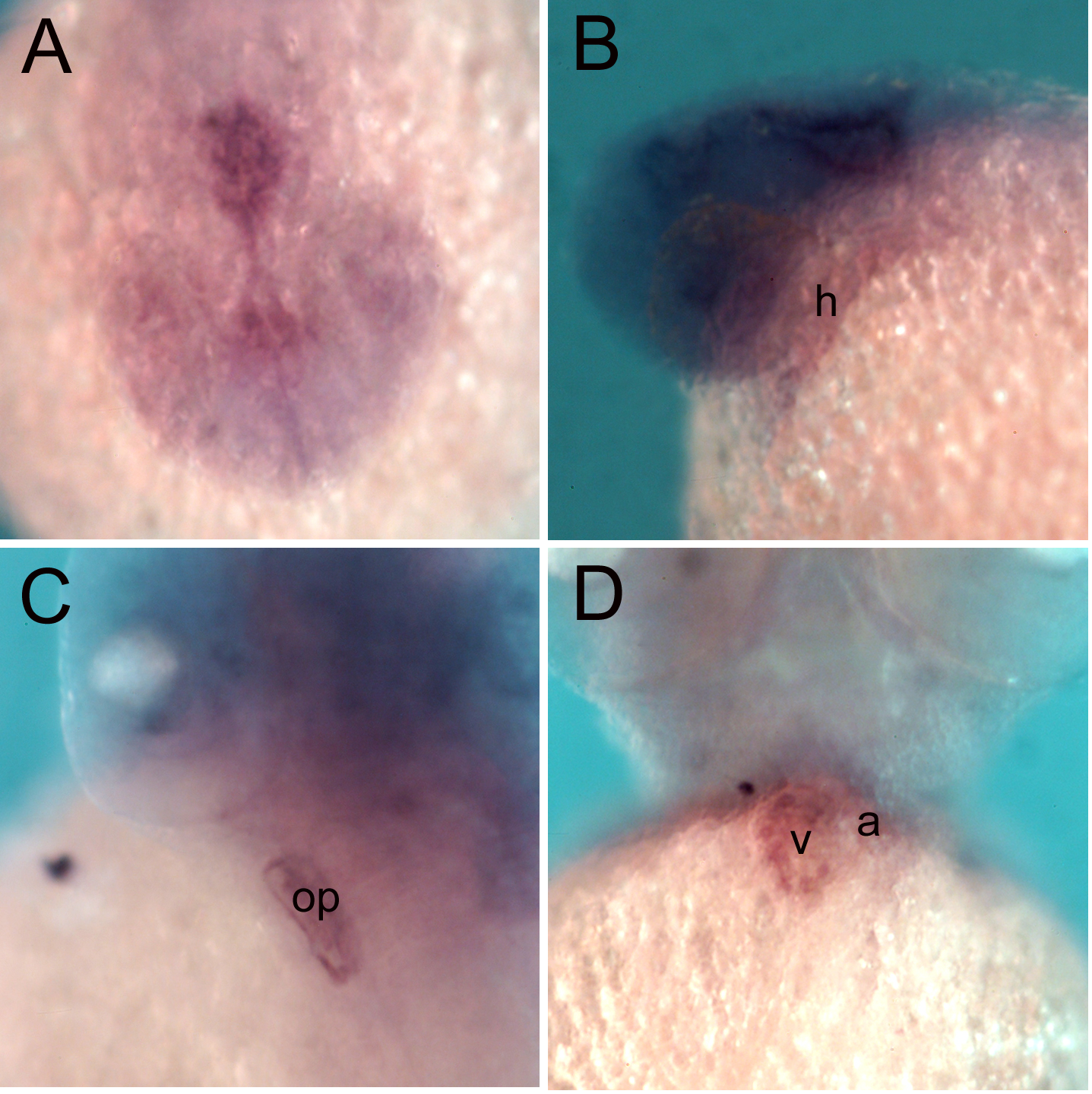

Supplement: Figure S3 — Expression pattern of zebrafish bmp10. Whole mount in situ hybridization of embryos at 24 hpf (A, B) and 60 hpf.(C, D). (A) dorsal view of the head; (B) lateral view; (C) lateral and dorsal view of the head and (D) ventral view of the heart. (A, B) Zebrafish bmp10 is expressed in the brain and the whole heart tube at 24 hpf. (C, D) At 60 hpf, bmp 10 is expressed in the myocardium of the heart chambers as well as in the otic vesicle. a, atrium; h, heart; op, otic vesicle; v, ventricle. (3.24 MB TIF) [file pone.0000398.s003.tif]

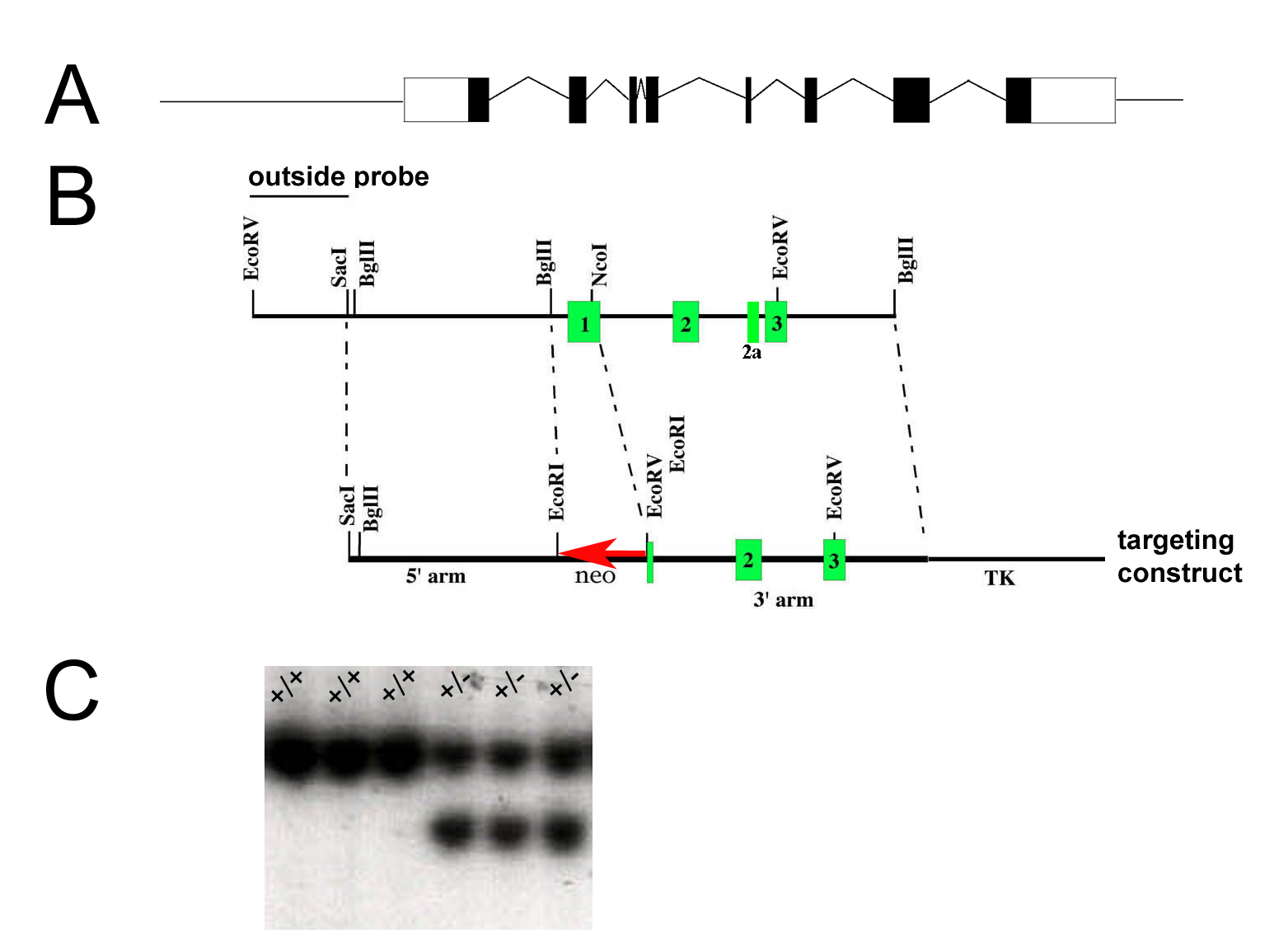

Supplement: Figure S4 — Targeting strategy to produce the Tbx3 null allele. (A) Schematic representation of genomic organization of the wild type mouse Tbx3 locus. (B) Diagram of the targeting strategy. Targeting construct showing the regions of homology and the site of insertion of the neo cassette in the Tbx3 locus. (C) Genomic southern analysis of EcoRV digested DNA of the ES cell clones. (0.43 MB TIF) [file pone.0000398.s004.tif]
