# Supplementary material for: Tbx2 and Tbx3 Regulate the Dynamics of Cell Proliferation during Heart Remodeling
Source: PLoS One. 2007 Apr 25;2(4):e398. doi: 10.1371/journal.pone.0000398 (PMC1851989; doi:10.1371/journal.pone.0000398)
Supplement: Methods S1 — (0.03 MB DOC) [file pone.0000398.s005.doc]

**Supplementary methods:**

Targeted disruption of the mouse *Tbx3* locus to produce the *Tbx3^neo^* allele by homologous recombination in embryonic stem (ES) cells was performed. A *loxP*-flanked *neo* selection cassette was inserted into the first exon of the *Tbx3* locus, using a targeting construct with an overall 5.3 Kb of homology (Fig. S 4 B). The targeting strategy resulted in the replacement of a *Bgl*II - *Nco*I fragment of 500bp located in the first exon and containing the ATG initiation site. This *neo* cassette contains multiple stop codons that prevent translation of *Tbx3* mRNA into protein and this generated allele was thus considered as a null mutated allele and called *Tbx3^neo^*. The linearized construct was electroporated in ES cells (J1 cells) and the colonies correctly targeted were selected by Southern analysis on *Eco*RV digests (Fig. S 4 C). Germ-line chimeras were generated by injection of targeted ES cells into C57BL/6 host blastocysts. Chimeras were mated with C57BL/6 females and the first progeny were confirmed to harbor the *Tbx3^neo^* allele by Southern analysis of *Eco*RV genomic digests, using a 5’ outside probe (Fig. S4 B) that binds a wild type 8.2 Kb fragment and a mutant 5.6 Kb fragment (not shown). Subsequently, mice and embryos were genotyped by PCR using the following primers: (1) 5’-CCAGTCATAGCCGAATAGCC-3’; (2) 5’- CTCAACTAAGACGCCTCCTG-3’; (3) 5’- GCAACAACAAAAGCGGAGCC-3’ that generate a 700 bp product from the wild type allele and a 400 bp product from the mutant allele.
